# Early Identification of Root Damages Caused by Western Corn Rootworms Using a Minimally Invasive Root Phenotyping Robot—MISIRoot

**DOI:** 10.3390/s23135995

**Published:** 2023-06-28

**Authors:** Zhihang Song, Tianzhang Zhao, Jian Jin

**Affiliations:** Department of Agricultural and Biological Engineering, Purdue University, 225 S University St., West Lafayette, IN 47907, USA; song399@purdue.edu (Z.S.); zhao770@purdue.edu (T.Z.)

**Keywords:** root phenotyping, western corn rootworm, RGB root imaging, in-situ, minimally invasive, deep convolution neural network, plant root traits measurement

## Abstract

Western corn rootworm (WCR) is one of the most devastating corn rootworm species in North America because of its ability to cause severe production loss and grain quality damage. To control the loss, it is important to identify the infection of WCR at an early stage. Because the root system is the earliest feeding source of the WCR at the larvae stage, assessing the direct damage in the root system is crucial to achieving early detection. Most of the current methods still necessitate uprooting the entire plant, which could cause permanent destruction and a loss of the original root’s structural information. To measure the root damages caused by WCR non-destructively, this study utilized MISIRoot, a minimally invasive and in situ automatic plant root phenotyping robot to collect not only high-resolution images but also 3D positions of the roots without uprooting. To identify roots in the images and to study how the damages were distributed in different types of roots, a deep convolution neural network model was trained to differentiate the relatively thick and thin roots. In addition, a color camera was used to capture the above-ground morphological features, such as the leaf color, plant height, and side-view leaf area. To check if the plant shoot had any visible symptoms in the inoculated group compared to the control group, several vegetation indices were calculated based on the RGB color. Additionally, the shoot morphological features were fed into a PLS-DA model to differentiate the two groups. Results showed that none of the above-ground features or models output a statistically significant difference between the two groups at the 95% confidence level. On the contrary, many of the root structural features measured using MISIRoot could successfully differentiate the two groups with the smallest *t*-test *p*-value of 1.5791 × 10^−6^. The promising outcomes were solid proof of the effectiveness of MISIRoot as a potential solution for identifying WCR infestations before the plant shoot showed significant symptoms.

## 1. Introduction

Maize (*Zea mays* L.) is an essential crop in the world due to its economic significance, versatility, and its critical role in sustainable agriculture. In the U.S. in 2022, the total yield was about 13.7 billion bushels, and the harvest area was 79.2 million acres [1]. However, pests can have a significant impact on corn production by reducing crop yields, increasing crop protection costs, and decreasing overall quality. The northern corn rootworm (*Diabrotica barberi* Smith & Lawrence) (NCR) and the western corn rootworm (*Diabrotica virgifera virgifera* LeConte) (WCR) are major economic pests of corn which cost U.S. producers about $1 billion annually in yield losses and input costs to control them [2]. Although the corn rootworm adults feed on corn leaves, silks, and kernels, which can cause potential yield loss by interfering with pollination, the most devastating period is the larvae stage because the larvae mainly feed on the corn roots [2]. The damaged corn roots cannot transfer water and nutrients effectively to the shoots, which can cause the plant to be more susceptible to diseases [3].

One of the common methods for assessing corn rootworm infestation in the root area involves cutting a 7-inch (18-cm) cube of soil around the base of the plant and lifting it out of the ground. The attached soil needs to be carefully removed from the roots and then the larvae can be examined either on a contrasting dark surface or by saturating the roots in salt water. This causes the larvae to float to the surface, making them easy to extract and count. To assess the damage caused by corn rootworm larvae in a certain field, the procedure needs to be repeated multiple times [4]. A group from Iowa State University developed another widely used method for evaluating root injury in corn plants [5]. This method also involves plant extracting and root washing. To rate the plants for injury, they must be at a growth stage where at least three nodes of roots are visible. While these methods are widely used worldwide, they typically involve a complete removal of corn plants from the soil during the sampling process and require a significant labor resource. However, minimizing any measurement’s effect on the roots is critical to avoid biasing noises and to ensure the possibility of having repeated measurements on the same plant. Extracting the entire plant may cause damage and alter the original root structure.

In recent years, several innovative methods have been developed to address some of the challenges in plant root phenotyping. For instance, a commonly applied approach called “minirhizotron” involves inserting a camera into a transparent tube pre-buried underneath the plants [6,7,8]. However, the transparent tubes are fixed underground, which renders them unsuitable for detecting damage caused by WCR larvae. This is because they cannot be relocated and have a limited field of view, restricting their effectiveness in monitoring the extent of the damage. Another common approach to conduct root phenotyping is to grow plants with certain types of Gellan gum with superior optical clarity [9] or other similar gel-based mediums [10]. This method provided the possibility of imaging the roots directly without physically interfering with them. However, the distinct physical characteristics of a gel-based medium may impede the mobility of WCR larvae and create an uninhabitable environment for them. Some other recent research for the three-dimensional reconstruction of the root architecture involves using technologies such as X-ray micro-computed tomography (μCT) [11], magnetic resonance imaging (MRI) [12,13], and ground penetrating radar (GPR) [14]. While these methods were capable of detecting roots non-destructively and sometimes with a fine resolution, their limitations are also evident. For instance, the working mechanisms, complexity, and size of μCT and MRI equipment limit their ability to conduct root phenotyping in field conditions or with field soil, especially when the soil moisture content is high [15]. Insufficient soil moisture content can impact the development of WCR larvae and may result in biased outcomes. The accuracy of the GPR is also limited by the relative water content in the soil and its resolution in detecting objects smaller than 4 cm [16].

This study utilized MISIRoot, an advanced and minimally invasive root phenotyping technology that enables in situ analysis of corn root structures in common soil environments [17]. This system is patented with the number US-20210005011-A1, and it offers several benefits, including its low cost, high-resolution imaging capabilities, and high accuracy in measuring the 3D structure of roots with a precision of up to 0.1 mm [18]. On top of the original robot, in this study, the MISIRoot system was equipped with the recent advanced deep convolution neural network model. All the MISIRoot images were automatically processed with high throughput and high accuracy. Combined with the 3D statistical analysis and color indices analysis, the system was able to provide versatile measurements of root traits and, thus, a better understanding of the roots’ growth behavior. A better understanding of how WCR damages the corn root at early stages could potentially assist in the development of better crop management strategies to mitigate the damage caused by these pests.

## 2. Materials and Methods

### 2.1. Experiment Design

**Figure 1:**
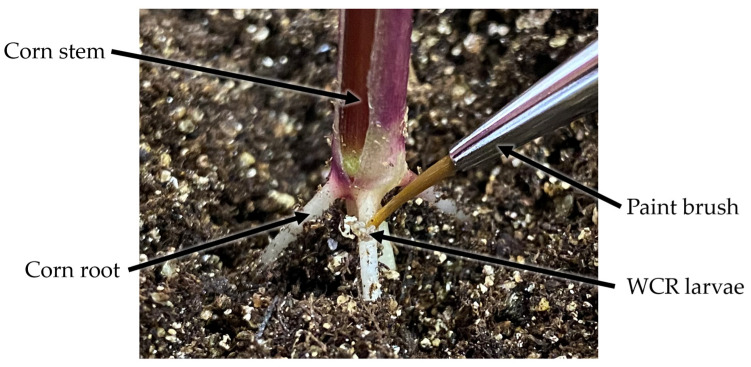
Close-up view of WCR larvae inoculated onto the crown root of a corn plant using a thin and soft miniature paintbrush.

A total of 32 corn plants were prepared in the Lily greenhouse at Purdue University, West Lafayette, USA. Half of the plants were randomly assigned to the inoculated group, while the remaining plants were assigned to the control group. A total of 10,000 WCR eggs were ordered and incubated in a closed growth chamber at 27 degrees Celsius and 70% relative humidity to facilitate hatching. Most of the WCR eggs started to hatch when the corn plants reached the V2 growth stage. A total of 100 WCR larvae were delivered to the crown root section of every plant in the inoculated group using a miniature paint brush as shown in Figure 1.

**Figure 2:**
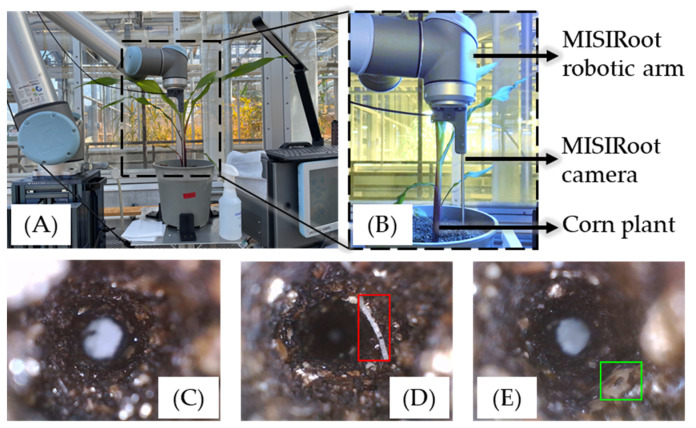
Image (**A**) shows the MISIRoot robot working on the collection of root images from one of the corn plants. Image (**B**) shows the basic MISIRoot structure with a close-up view. Images (**C**–**E**) are examples of MISIRoot images, in which (**C**) shows an image without the root being detected inside, (**D**) shows an image with a thin root detected and labeled in a red rectangle, and (**E**) shows an image with a thick root detected and labeled in a green rectangle.

After the inoculation process, the roots that were inoculated by the larvae were gently covered with soil. Nylon nets were also placed over the pots to prevent the larvae from escaping from the pot. The WCR larvae will undergo metamorphosis and develop into beetles in approximately 3 to 4 weeks. Since adult WCR pests have strong motility that may result in uncontrollable contamination of the environment, the sampling process was initiated 15 days after the inoculation, before the pests turned into adults as shown in Figure 2.

Before the MISIRoot system, side-view images of the corn plants were captured using a Nikon D5300 camera, with the aperture set to f/9, exposure time set to 1/60 s, and ISO set to 200. During the data collection process, a sampling area with a diameter and depth of 100 mm was designated, resulting in the generation of 130 sampling tunnels around each plant. Each tunnel contained 35 viewpoints, leading to the acquisition of 4550 images per plant. Once completed, all plant materials, including the soil and pots, were decontaminated using an autoclave. After sterilization, the roots were carefully extracted and washed for visual examination and side-view imaging.

### 2.2. Shoot Data Analysis

**Figure 3:**
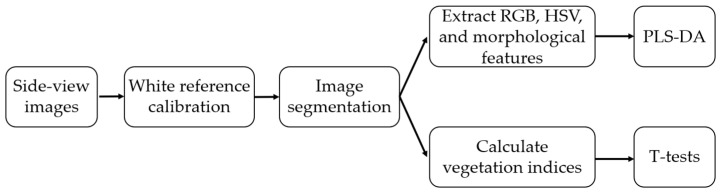
Working flow to analyze the side-view RGB images of the corn plants.

This project aimed at demonstrating that MISIRoot was capable of detecting the WCR damage to corn plant roots before symptoms of lodging or nutrient deficiency become visible in the shoot section. To achieve this, two different approaches were used to analyze the side-view RGB images of the corn plants. In the first approach, the color information and the morphological features of the plant leaves were extracted as the input of a Partial Least Squares-Discriminant Analysis (PLS-DA) modeling process, which would be able to tell if the two treatment groups could be well separated. The PLS-DA model was specifically selected because it has been extensively utilized in plant phenotyping applications to identify plant stress thanks to its exceptional ability to manage high-dimensional data and mitigate the issues of multicollinearity [19,20]. The second approach involved calculating various vegetation indices. The working flow for both approaches is illustrated in Figure 3 below.

**Figure 4:**
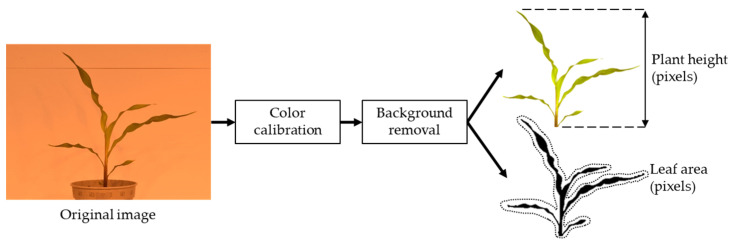
Working flow to extract the morphological features of the corn plants.

Before applying both approaches, the images were calibrated for ambient light variations using the white reference board positioned behind the corn plant. The corn plants were calibrated and segmented from the image to remove any ambient light and background noises. After segmentation, the average values for red (R), green (G), and blue (B) were extracted from the remaining pixels. To facilitate analysis, the RGB color space was then converted to the HSV (Hue, Saturation, Value) color space, which separates the brightness component (value) from the chromatic components (hue and saturation). The HSV color space is commonly used in machine vision and image processing applications, including plant phenotyping [21], as it enables the separation of brightness from color, making it particularly useful for analyzing images captured under varying lighting conditions. Moreover, as shown in Figure 4, two morphological features were extracted from the segmented images. The number of pixels remaining after segmentation was counted as an indicator to estimate the leaf area (biomass), and the height of the plants was assessed by calculating the distance between the top pixel and the bottom pixel.

In the first approach, partial least squares discriminant analysis (PLS-DA) was conducted by integrating the RGB and HSV color information with leaf area and plant height. Monte Carlo cross-validation (MCCV) was used to randomly partition the data into training and testing sets in a 70:30 ratio for 1000 iterations. For the second approach, 10 vegetation indices based on RGB color space were utilized as shown in Table 1. These indices were used to demonstrate the changes in visible light spectral information corresponding to different chlorophyll contents [22], which can be used to detect different nutrient stresses of the plants. These indices were applied to the segmented images, and the results from the control group and the inoculated group were compared using two-tailed *t*-tests.

### 2.3. Root Data Analysis

The data obtained from the MISIRoot system can be divided into two parts: raw images and corresponding 3D coordinates of each image.

**Figure 5:**
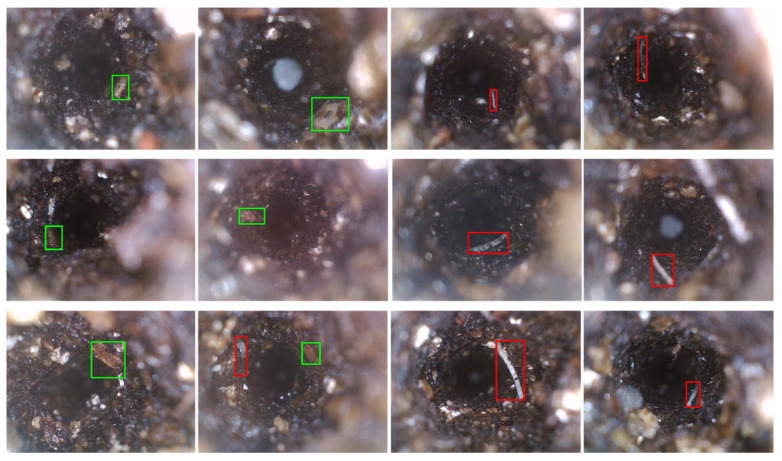
Examples of the CNN object detection model predictions using bounding boxes. Red bounding boxes meant thin roots. Green bounding boxes meant thick roots.

A total of 135 images in which the root is visible were selected for training a deep convolutional neural network model for high-throughput image processing. In each image, every visible root section was manually labeled with square-shape bounding boxes indicating both the existence and the types of roots found in the image. Types of the root were grouped into “thin” and “thick” categories based on the thickness and color of the root detected in the image. When the root was wider than 60 pixels in the image and had a slight yellow or brown color, it would be labeled as a “thick” root, otherwise, it would be labeled as a “thin” root. A total of 95 labeled raw images (70%) were randomly chosen for training, and the remaining 40 images (30%) for validation. Before being fed to the model, the data was augmented with random horizontal or vertical flips based on a 50% random chance. The base model structure selected for training was Faster R-CNN with a ResNet-50-FPN backbone [32,33]. The training optimizer was SGD with the start learning rate at 0.005. After 70 epochs of training, the loss was converged. The final model was selected at the 100 epochs of training with a mean average precision of 0.94. Some examples of the model predictions are shown in Figure 5. If a root was detected and marked with a red bounding box, it indicated a thin root found in the image. A green bounding box means a think root.

**Figure 6:**
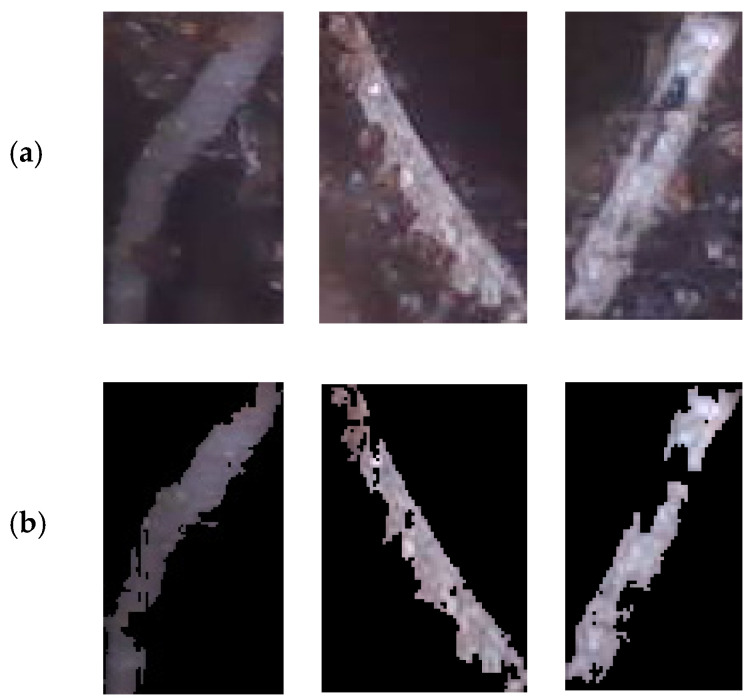
Example images of root segmentation. (**a**) Images before segmentation. (**b**) Images after segmentation by marking the background with black color.

After processing with the deep learning model, the data can be further categorized into four main parts: number of detected roots for each plant, color information of the detected roots, classification of root thickness, and coordinates for each identified root. For effective utilization of the data, it is crucial to obtain accurate color information on the roots. A clean segmentation that eliminates soil background, soil particles on the roots, and any specular reflectance present on the roots is a crucial step in extracting the color information, as illustrated in Figure 6.

**Figure 7:**
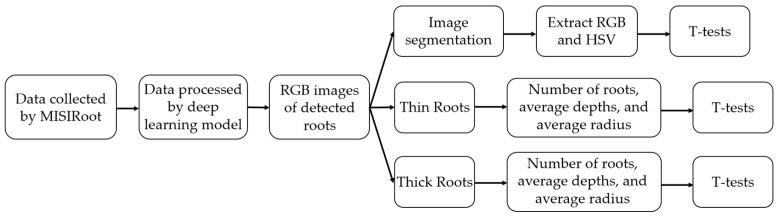
A working flow that shows how the data collected by MISIRoot were processed.

The average R, G, and B values, as well as the H, S, and V values of the remaining pixels from the segmented images were calculated and separated into two groups based on the thickness classification of the roots. *t*-tests were then conducted to ascertain the presence of any statistically significant differences between the two groups for each of these values. Further analysis was conducted on the classification of root thickness, as well as the coordinates of the detected roots The coordinates can be used to determine the depth, which is the distance below the surface, and radius, which is the distance from the plant’s main stem, for each identified root. The purpose of this analysis was to gain a better understanding of the distribution of roots and the extent of damage inflicted upon them. The working flow for analyzing the data collected by the MISIRoot system is illustrated in Figure 7 below.

## 3. Results

### 3.1. Data Analysis

To determine whether there were significant differences between the above-ground portions of two groups of corn plants, the first approach involved constructing a PLS-DA model using the information extracted from the side-view images. Following cross-validation, the average training accuracy and average testing accuracy were found to be 48.69 percent and 50.27 percent, respectively. While the testing accuracy is higher than random guessing, it is still considered relatively low and indicates that the model has limited discriminative power.

**Figure 8:**
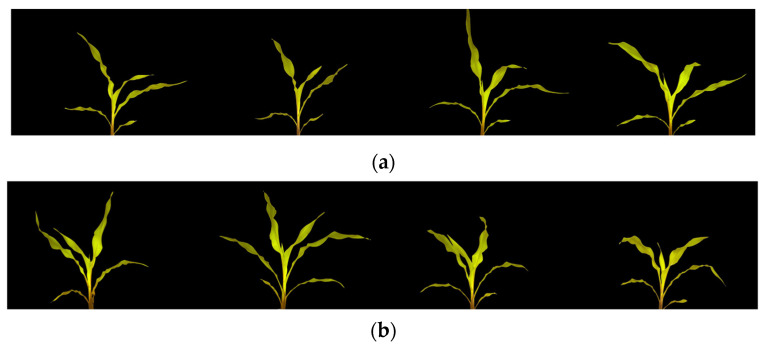
Examples of calibrated and segmented images of corn plants for the control group (**a**) and the inoculated group (**b**).

The second approach employed 10 vegetation indices to identify areas where the two groups exhibit discernible differences in nutrient stress. The *p*-values in Table 2 indicate that none of the values are smaller than 0.05, which suggests that there is no significant difference between the two groups. Additionally, as illustrated in Figure 8 below, there is no discernible visual difference between the two groups from the calibrated and segmented images.

### 3.2. Data Analysis for High-Resolution Images Collected by MISIRoot

The outcomes obtained from high-resolution RGB images collected by the MISIRoot are presented in Table 3. Table 3 shows that after segmentation, the *p*-values for the R, G, B, and V values for thin roots were found to be smaller than 0.05, indicating that there were statistically significant differences between the inoculated and control groups for these values.

**Figure 9:**
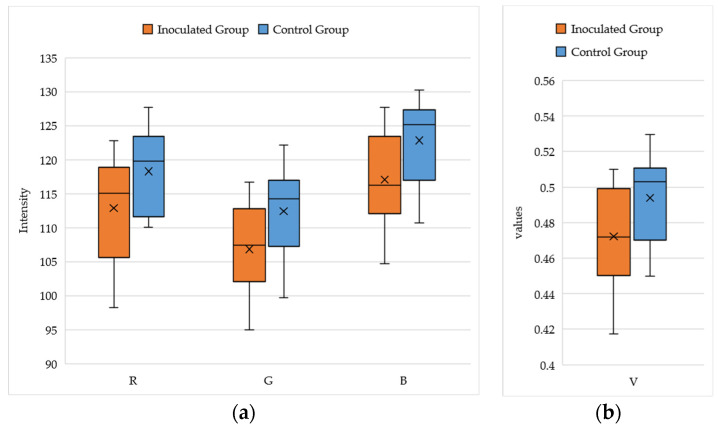
The RGB (**a**) and V (**b**) values of the roots found in the images from both the inoculated group and the control group.

**Figure 10:**
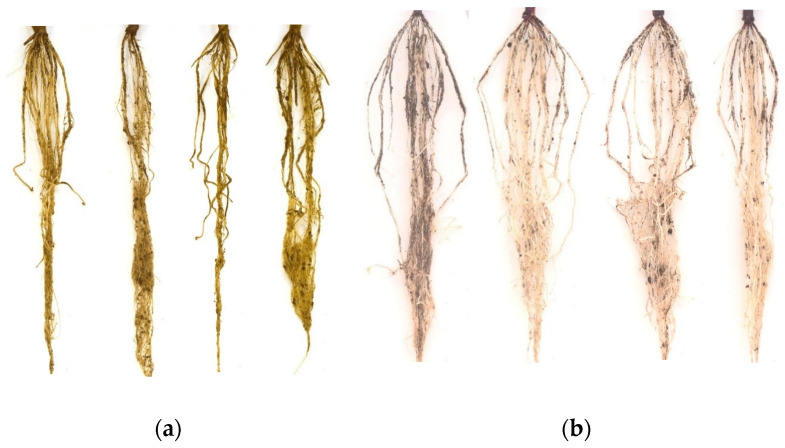
Example images of the extracted roots. Roots from the inoculated group are shown in (**a**). Roots from the control group are shown in (**b**).

The results presented in Figure 9 indicate that the R, G, B, and V values of the control group are higher compared to those of the inoculated group, suggesting that the roots of the control group have a greater intensity or brightness. This observation is further supported by the images presented in Figure 10, which were obtained after sterilization and calibrated using the white reference board. The roots of the inoculated plants exhibit a darker color compared to the roots of the control plants.

### 3.3. Data Analysis for Data Processed by Deep Learning Model

Further analysis was conducted on the number of roots detected, as well as the average depth and radius of the roots. The roots were categorized into two groups based on their thickness, resulting in a total of six *t*-tests being performed. Table 4 indicates that the *p*-values for the number of thin roots detected, and the average depth of thin roots exhibit significant differences between the two groups, as they are substantially lower than 0.05. The thin roots showed significant differences between the control and inoculated groups, whereas no significant differences were observed for the thick roots.

**Figure 11:**
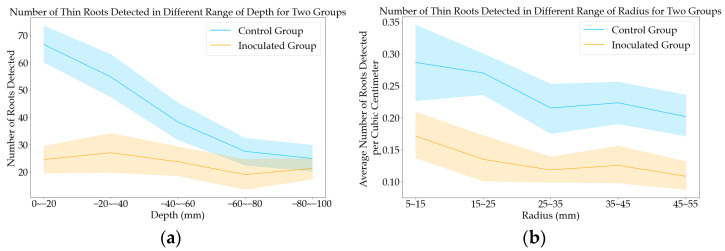
(**a**) The average number of detected thin roots across the depth for two groups. (**b**) The average number of detected thin roots across the radius for two groups. Figures (**a**,**b**) utilize blue and orange colors to represent the control and inoculated groups, respectively. The line indicates the average number of detected roots, while the shaded region represents one standard deviation above and below the mean.

To gain a deeper understanding of the results, the number of detected thin roots was analyzed with their depth and radius. The sampling depth for this project was 100 mm and the sampling radius for this project was 50 mm. The depth was divided into five regions, with each region covering a depth of 20 mm. The radius was divided into five concentric circles, with each circle covering approximately 10 mm, as shown in Figure 11a. Figure 11a depicts the average number of thin roots detected across the entire depth range. The discrepancy in the number of detected roots between the two groups diminishes as depth increases, suggesting that the roots in the shallower region experience the most significant damage. Figure 11b shows the average number of thin roots detected per cubic centimeter across the entire radius range. In this study, cubic centimeters were used to quantify the distribution of roots instead of simply using the number of roots. This approach provided a more accurate representation of the root distribution, as the number of sampling points in the outer radius was significantly greater than in the inner radius. Figure 11b illustrates the average number of thin roots per cubic centimeter for healthy and inoculated plants across all radius ranges. The results show that the healthy plants have a higher average number of thin roots per cubic centimeter than the inoculated plants, indicating that the damage caused by corn rootworm larvae is widespread and affects the entire root system of the plant.

### 3.4. Results Comparison between Three Different Approaches

**Figure 12:**
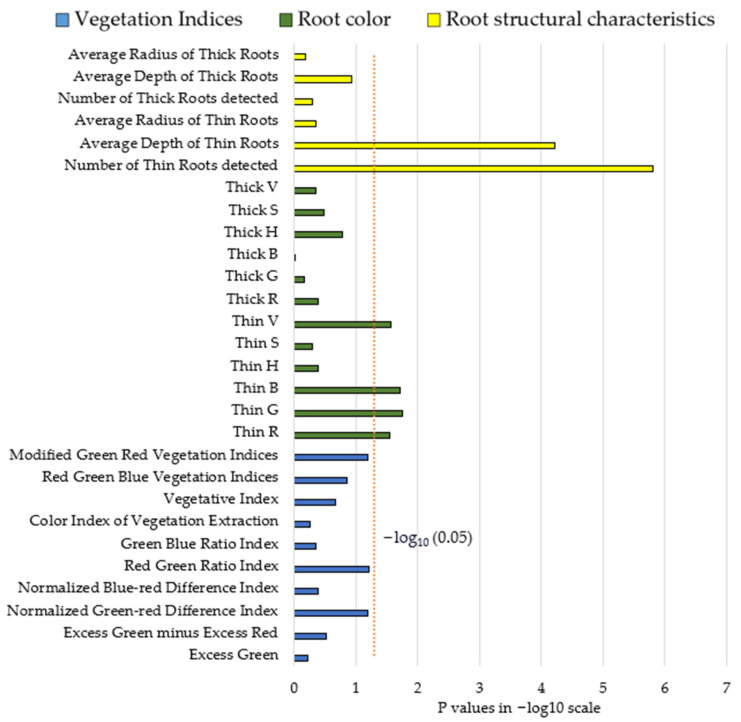
A comparison of all *p*-values from three different approaches on a −log10 scale.

The results were compared to provide a more direct way of interpreting the differences between data collected by the DLSR camera from the shoot section and data collected by the MISIRoot. Figure 12 displays the comparison of *p*-values from all tests on a −log_10_ scale and the orange dashed line represents a *p*-value of 0.05. While none of the *p*-values for vegetation indices were smaller than 0.05, certain *p*-values for data collected by the MISIRoot system were found to be significant at this threshold. While color information for thin roots exhibits the capability to distinguish between the two groups, the average depth of thin roots and the number of thin roots detected yield the most promising results, as evidenced by the significant *p*-values of 6.0481 × 10^−5^ and 1.5791 × 10^−6^, respectively.

## 4. Discussion

Most of the current WCR detection methods necessitate the complete destruction of plants and rely on the emergence of severe symptoms, such as “gooseneck lodging” in the shoot section [4]. However, these methods are time-consuming, demand significant human labor, and do not facilitate early-stage control in a timely manner.

This paper focuses on detecting damage caused by WCRs at early growth stages using an innovative root phenotyping system called MISIRoot. The larvae were specifically placed on the crown root section of the corn plants, resulting in more pronounced damage occurring in the shallower region. Moreover, root damage in the shallower region inhibits the development of finer roots. Because MISIRoot is capable of estimating the root density distribution, the thin root damaged by WCR, which primarily infests root hairs and outer root tissue [4], can be successfully differentiated from the healthy roots shown in Figure 11. Additionally, the high-resolution RGB camera equipped using the MISIRoot system ensures the success of detecting the appearance of brown root tips damaged by WCR [4].

Moreover, the unique design and workflow of the MISIRoot system make it suitable for field applications. It can be conveniently transported by ground-based vehicles or installed on center-pivot irrigation systems. While the current design of the system can benefit from improved throughput, the challenges can be addressed by incorporating additional cameras. The deep learning model achieved a high accuracy with the current dataset and experiment setup. Some limitations may exist when dealing with different soil colors or different plant root stress. The model performance could also be affected by some other factors, such as the images being blurred or blocked by the soil. The model can be further trained with newly labeled data coming from the new environment. Nonetheless, it is important to acknowledge that the sample size was limited due to resource constraints. Conducting additional experiments or adding more replicates can enhance statistical power in *t*-tests. It is also worth noting that certain factors, such as the number of larvae and the time of inoculation, were carefully controlled for experimental purposes. Challenges may arise in future applications when trying to determine the ideal detection time window. Numerous other factors, including environmental and soil conditions, must be considered to determine the appropriate timing for implementing the MISIRoot system in similar tasks.

## 5. Conclusions and Future Work

Through the analysis of side-view images of corn plants and data collected by the MISIRoot system, this study demonstrated the system’s ability to detect damages caused by WCR larvae to the corn root system before visible differences appeared in the above-ground portion of the plants. The data collected with MISIRoot was first processed using a CNN model, which labels the detected root segments in the images. The root color exhibited noticeable differences between the inoculated and control groups, which was confirmed by visual examination after the roots were extracted. Better separation between the treatment groups was achieved by integrating additional features, such as the number of roots detected, the average depth and radius of the detected roots, and the thickness of the roots. Overall, the MISIRoot system has demonstrated its potential to revolutionize the way to assess WCR infestation in an affordable and non-destructive manner.

Future research can involve time series analysis to further advance our understanding of the damages caused by WCR larvae and refine the detection protocol. It is possible to capture the damage development by repeating the measurements over an extended period of time. Additionally, such analysis can facilitate a comparison between the MISIRoot system and other established methods, helping determine whether the MISIRoot system is capable of detecting damages earlier than existing approaches. Additionally, the throughput of the system can be further improved by adding more imaging heads and the quality of the images can also be further enhanced by equipping more advanced cameras.

## Figures and Tables

**Table 1:** The formula for calculating vegetation indices based on the corn plant above-ground tissue images.

Index	Formula	References
Excess Green	2×G−R−B	[23]
Excess Green minus Excess Red	ExG−ExR	[24]
Normalized Green-red Difference Index	(G−R)/(G+R)	[25]
Normalized Blue-red Difference Index	(G−B)/(G+B)	[25]
Red Green Ratio Index	R/G	[26]
Green Blue Ratio Index	B/G	[27]
Color Index of Vegetation Extraction	0.441×R−0.811×G+0.385×B+18.78745	[28]
Vegetative Index	$G/\left(R^{a}\times B^{1-a}\right),\;\mathrm{where}\;a=0.667$	[29]
Red Green Blue Vegetation Indices	$\left(G^{2}-B\times R\right)/\left(G^{2}+B\times R\right)$	[30]
Modified Green Red Vegetation Indices	$\left(G^{2}-R^{2}\right)/\left(G^{2}+R^{2}\right)$	[31]

**Table 2:** *p*-values of vegetation indices for *t*-tests between the inoculated group and the control group.

Index	*p*-Values
Excess Green	0.5998
Excess Green minus Excess Red	0.3050
Normalized Green-red Difference Index	0.0652
Normalized Blue-red Difference Index	0.4194
Red Green Ratio Index	0.0633
Green Blue Ratio Index	0.4460
Color Index of Vegetation Extraction	0.5593
Vegetative Index	0.2143
Red Green Blue Vegetation Indices	0.1399
Modified Green Red Vegetation Indices	0.0652

**Table 3:** *t*-test *p*-values for root color between the inoculated group and the control group, with the null hypothesis stating that the two groups have the same mean value.

	R	G	B	H	S	V
*p*-values for thin roots	0.0289	0.0176	0.0196	0.4149	0.5186	0.0268
*p*-values for thick roots	0.4108	0.6796	0.9694	0.1699	0.3332	0.4517

**Table 4:** *t*-test *p*-values for the number of roots, average depth, and average radius of detected roots.

	Number of Roots Detected	Average Depth of Roots	Average Radius of Roots
Thin	1.5791 × 10^−6^	6.0481 × 10^−5^	0.4545
Thick	0.5094	0.1178	0.6605

## Data Availability

The data presented in this study are available on request from the corresponding author. The data are not publicly available due to privacy.
